# A Survey of Context-Aware Access Control Mechanisms for Cloud and Fog Networks: Taxonomy and Open Research Issues

**DOI:** 10.3390/s20092464

**Published:** 2020-04-27

**Authors:** A. S. M. Kayes, Rudri Kalaria, Iqbal H. Sarker, Md. Saiful Islam, Paul A. Watters, Alex Ng, Mohammad Hammoudeh, Shahriar Badsha, Indika Kumara

**Affiliations:** 1 Department of Computer Science and Information Technology, La Trobe University, Melbourne, VIC 3086, Australia; r.kalaria@latrobe.edu.au (R.K.); p.watters@latrobe.edu.au (P.A.W.); alex.ng@latrobe.edu.au (A.N.); 2 Swinburne University of Technology, Melbourne, VIC 3122, Australia; iqbal.sarker.cse@gmail.com; 3 Griffith University, Gold Coast, QLD 4215 Australia; saiful.islam@griffith.edu.au; 4 Manchester Metropolitan University, All Saints, Manchester M15 6BH, UK; m.hammoudeh@mmu.ac.uk; 5 University of Nevada, Reno, NV 89557, USA; sbadsha@unr.edu; 6 Jheronimus Academy of Data Science, Sint Janssingel 92, 5211 DA’s-Hertogenbosch, The Netherlands; i.p.k.weerasingha.dewage@tue.nl

**Keywords:** cloud-based data resources, Internet of things, privacy protection, security, centralized environments, context-aware access control, fog-based access control, contextual conditions, authorization, decentralized environments

## Abstract

Over the last few decades, the proliferation of the Internet of Things (IoT) has produced an overwhelming flow of data and services, which has shifted the access control paradigm from a fixed desktop environment to dynamic cloud environments. Fog computing is associated with a new access control paradigm to reduce the overhead costs by moving the execution of application logic from the centre of the cloud data sources to the periphery of the IoT-oriented sensor networks. Indeed, accessing information and data resources from a variety of IoT sources has been plagued with inherent problems such as data heterogeneity, privacy, security and computational overheads. This paper presents an extensive survey of security, privacy and access control research, while highlighting several specific concerns in a wide range of contextual conditions (e.g., spatial, temporal and environmental contexts) which are gaining a lot of momentum in the area of industrial sensor and cloud networks. We present different taxonomies, such as contextual conditions and authorization models, based on the key issues in this area and discuss the existing context-sensitive access control approaches to tackle the aforementioned issues. With the aim of reducing administrative and computational overheads in the IoT sensor networks, we propose a new generation of Fog-Based Context-Aware Access Control (FB-CAAC) framework, combining the benefits of the cloud, IoT and context-aware computing; and ensuring proper access control and security at the edge of the end-devices. Our goal is not only to control context-sensitive access to data resources in the cloud, but also to move the execution of an application logic from the cloud-level to an intermediary-level where necessary, through adding computational nodes at the edge of the IoT sensor network. A discussion of some open research issues pertaining to context-sensitive access control to data resources is provided, including several real-world case studies. We conclude the paper with an in-depth analysis of the research challenges that have not been adequately addressed in the literature and highlight directions for future work that has not been well aligned with currently available research.

## 1. Introduction

Computer security is a very complex phenomenon in today’s environments, like the cloud-based Internet of things (IoTs) [1], where different users interact with each other, and the infection of one creates risk for another. Access control [2] is a fundamental aspect of computer security that is directly contributing to the traditional privacy and security principles of confidentiality, integrity and availability (CIA) [3]. However, these CIA security characteristics are very restricted in such dynamic environments. These IoTs and smart spaces provide the seamless interconnection of billions of devices and sensors over the Internet [4,5].

Users involved in the IoT-based scenarios often need to access data and information resources beyond that which may normally be associated with their given roles. This has created the need for a new approach to context-aware access control and the concomitant triggering of relevant contexts [6,7,8] using information provided through IoT connected devices. For example, a paramedic is allowed to dynamically activate and play an “emergency-paramedic” role to provide advanced treatments to a patient at the “scene of an accident” through utilizing IoT-based resources, when the patient’s current health condition is “critical”. These dynamic contextual conditions can be effectively derived from the IoT devices and relevant smart environments in order to manage such critical situations.

In today’s dynamic environments, new opportunities have been created for the management of critical situations utilizing the IoTs and smart spaces. However, one of the difficulties in providing such IoT-based services or accessing IoT-based resources is that access will often be needed by teams or users at the critical events, where access to data and information resources is usually restricted by means of their normal roles that are associated with relevant contextual conditions. Therefore, one of the big challenges is that the security mechanisms would have the capability of making access control decisions based on the relevant contextual conditions and consequently would re-evaluate the decisions when there are dynamic changes to the context.

We have been encountering rapid changes in computing technologies in the last couple of decades and many organizations have been seeking appropriate access control mechanisms to dynamically control context-sensitive access to data resources from distributed cloud environments. Due to this paradigm shift from a fixed desktop environment to dynamic environments [9], the role of dynamically changing contextual conditions has gained great importance for context-sensitive decision making. The way to cope with this paradigm is to develop a new generation of access control mechanism to manage and control context-sensitive access to information and data resources in today’s dynamic world and thus demanding for further studies on many aspects of CAAC mechanisms and approaches. According to the traditional CAAC approaches [6,10], context (we use the term context or contextual condition interchangeably in this paper) means information about the state of an access control-specific entity or an associated relationship between entities. An access control-specific entity can be a user, data resource, or their surrounding environments.

### 1.1. The Background

Over the last few decades, different research communities have introduced significant numbers of context-sensitive access control approaches and frameworks that differ in their context models, policy models, and reasoning capabilities. Several Role-Based Access Control (RBAC) models [11,12,13,14] have been proposed in the literature, incorporating the dynamically changing contextual conditions (e.g., user and resource-centric information) into the RBAC policies. Like spatial and temporal RBAC approaches [15,16,17,18], these context-sensitive RBAC approaches are mostly domain-specific and consider specific types of contextual conditions.

In recent years, we have introduced a family of Context-Aware role-based Access Control (CAAC) approaches [6,7,8,10] to facilitate access control to data resources based on the wide range of contextual conditions. In our CAAC approaches, we have considered the general context information (e.g., location, request time, profile information) [6,19], the relationship context information (e.g., relationship type, strength and granularity level) [20] and the situational context information (e.g., research purpose and treatment purpose to access resources) [10,21].

We have introduced a new approach to context-sensitive access control for the specification and activation of dynamic contextual roles based on the relevant contextual conditions [8]. Recently, we have introduced a new generation of context-sensitive approach to access data from multiple sources, incorporating the contextual conditions [22]. Extending our initial access control model proposed in [22], we recently have proposed a data view model to provide an integrated result to the users, dealing with the privacy requirements of the associated stakeholders [23]. These contextual conditions are mostly obtained by exploiting the classical crisp sets and are needed to be incorporated into the CAAC policies [24,25]. However, there are some other conditions that cannot be obtained directly from such crisp sets. As such, we have exploited the fuzzy sets and introduced a fuzzy context information system [7] by utilizing the basic fuzzy model [26].

Looking at the existing CAAC approaches, these solutions extensively have been used to access data and information resources from centralized sources [27,28]. These approaches do not provide adequate functionalities to access required data sets from distributed environments (e.g., distributed cloud centres). Different data integration techniques have been developed over the last few decades to collate data from multiple sources, such as schema matching [29,30], entity resolution [31], record linkage [32,33], data fusion [34], global view [35], and ontology-based [36,37] approaches. These data integration techniques and approaches mostly have been used to map original sources of data (i.e., different schema) and result in global data models.

Due to the technological advancements in the Internet-based environment, currently, different stakeholders have been seeking access control mechanisms to access data from many distributed sources. The current IoT cloud platforms [38] seek a new form of context-sensitive access control model for understanding mechanisms of controlling data and information resources from different Big Data sources [39]. Perera et al. [40] have conducted a survey addressing a range of models, applications, systems and middleware solutions related to IoT and context-awareness. In the study, they have evaluated the commercial and research solutions proposed in the field of context-aware computing and IoT perspectives over a decade, starting from 2001 to 2011.

The integration of data directly from distributed sources raises semantic namespace and latency problems [41,42] due to the lack of semantics and cloud-based services. The richer semantics of the data model is still needed to resolve the semantic namespace problem, dealing with the distributed nature of such big data sets. However, the latter is forcing the organizations to overcome the latency issue by adding intermediary computational nodes at the edges of the networks [43].

In recent years, different fog computing models have been introduced to reduce the latency and processing overheads involved in managing and accessing data resources from the cloud sources towards the edge of the end-devices (e.g., [44,45,46,47]). These fog nodes usually provide intermediary computation and networking services between the end-users and the traditional cloud data servers. Over the last few years, a number of fog-based access control approaches have been proposed in the literature (e.g., [48,49,50,51]).

Zaghdoudi et al. [48] propose a generic and scalable access control model for fog computing with low overhead, considering the information about the subjects, objects and operations as contexts. In [49], the authors present a recent study on intelligent transport systems utilizing fog computing and identify the corresponding fog-based access control issues. Both research works have been concerned with several important requirements of the fog-based access control models, such as context-awareness and distributed architecture. They also discuss the decentralization of authority from a single administrative location to other locations.

Recently, Yu et al. [50] and Zhang et al. [51] propose the fog-based access control models in order to provide a way to securely share data resources along with the benefits of encryption and decryption methods. These existing fog-based access control mechanisms can be used to build access control solutions to access data and information resources from multiple environments. However, they are not truly context-aware and robust enough to support context-sensitive access control to data resources from distributed cloud sources. One of the big challenges is the lack of access control mechanisms to control and manage data from distributed environments [52]. Another challenge is the lack of privacy along with maintaining the trust of users [53,54].

### 1.2. The Contributions

Based on the above-mentioned investigations, the main contributions of this work are listed as follows.

1. We discuss the research challenges with a literature review whose main purpose is to identify the relevant contextual conditions for context-sensitive access to data resources in the cloud and fog networks.
2. These dynamically changing conditions are further analyzed to propose a new generation of Fog-Based Context-Aware Access Control (FB-CAAC) model, combining the benefits of the cloud, IoT and context-aware computing along with traditional data integration solutions. Towards this end, we demonstrate different taxonomies and an empirical analysis of existing context-aware access control mechanisms.
3. We highlight possible directions for future work that have not been well covered in current state-of-the-art context-aware access control research.

This paper is therefore intended to be a starting point to address the associated research issues of building required CAAC mechanisms to access information and data resources from distributed cloud sources, by taking into advantage of fog, IoT and context-aware computing. Towards this end, this paper presents an extensive survey of numerous approaches and mechanisms of context-aware access control which are gaining a lot of momentum in both industrial and scientific communities. We discuss the advantages and disadvantages of each mechanism and its suitability to support context-aware access control to cloud and fog networks. This survey is very important to the potential industrial and research communities by knowing the requirements of the CAAC applications and how they manage and control IoT data in the cloud.

### 1.3. The Motivation and Scope of the Survey

In this section, our goal is to motivate the study of context-aware access control for the management of applications in today’s interconnected world, especially in the cloud, fog and IoT environments. We also present the scope of our survey.

**Figure 1:**
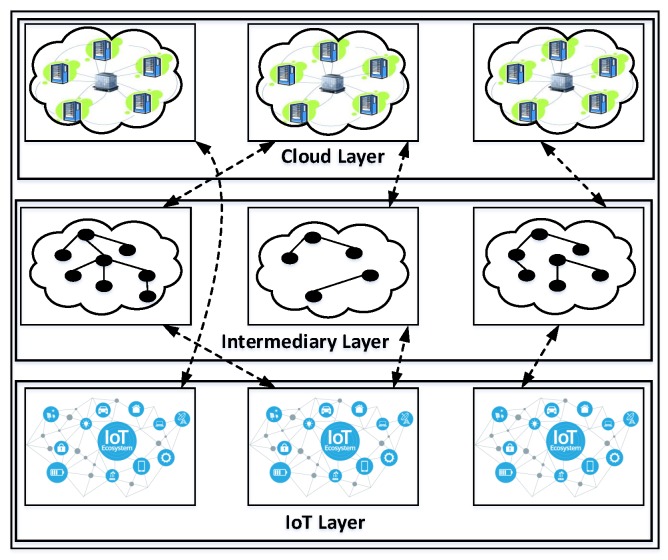
The relationship chain between different computing paradigms.

We are currently living in the era of big data, with the involvement of distributed clouds for storage. We illustrate the relationship chain among Cloud, Fog and IoT sources/applications and separate these into three layers in Figure 1. These three layers provide different levels of abstraction and granularity of access control to data from multiple sources. Fog computing is a new computing paradigm introduced recently [45], which extends the cloud computing paradigm to the periphery of the networks in order to reduce the latency and processing overheads involved in managing and accessing cloud-based data resources.

One of the fundamental challenges here is to control different types of information and data resources from distributed sources, provide access to users, and consequently maintain multiple levels of access control both in the cloud layer and to the edge of the network in the end-user layer. In this study, we mainly cover the existing traditional access control and context-aware access control mechanisms that are relevant within the scope of this survey, especially which are applicable in today’s cloud and fog networks. Table 1 includes the scope of this survey (from ’S1’ to ’S8’).

### 1.4. The Outline

The rest of this paper is organized as follows. The motivation and scope of the survey are discussed in Section 1. In Section 2 and Section 3, we briefly introduce the traditional access control and the context-aware access control literature respectively. We discuss the current state-of-the-art CAAC frameworks for centralized networks in Section 4. In Section 5, we discuss the access control and privacy-preserving mechanisms for decentralized networks. In Section 4 and Section 5, we also provide a comparative analysis on different access control mechanisms. In Section 6, we present an in-depth analysis of the open research issues and propose a new generation of fog-based CAAC framework for cloud and IoT networks. Finally, we conclude the paper in Section 7.

## 2. The Traditional Access Control

This section includes the background of access control, including the traditional security mechanisms.

Access control [2] is a classic security model that is the cornerstone of any security and privacy protection mechanisms to support data access from different environments, to protect unauthorized access according to a security policy, and to verify whether a user is allowed to carry out a specific action on the information and data resources.

### 2.1. Discretionary Access Control

Access control technology has a long history that started in the late 1960s. It was first introduced by Lampson in 1971 [55], who proposed a formal and mathematical description of a basic access control model named access control matrix. In 1983, access control technology took a significant step forward when the U.S. Department of Defence (DoD) defined Discretionary Access Control (DAC) [56]. In general, access control models can be classified as discretionary and non-discretionary access control. DAC is defined by the trusted computer system evaluation criteria (TCSEC) [56] as a means of restricting access to objects based on the identity of subjects and/or groups to which they belong.

The following are the basic building blocks of a DAC system: Access Control List [57] and MAC Policy.

- Access Control List: An access control list contains entries for the subjects, which describe the operations that the subjects can execute on the given objects. For example, a file has an access control list that contains (Mary, read), which gives Mary permission to read the file.
- DAC Policy: In the DAC-based policy, the object owners specify who can access what objects through access control policies.

Like access control list, the capability-based access control model [2] is also a DAC-based access control solution, where a row centric view has been introduced and each row can be seen as a capability. The subjects each have capability lists to access the given objects. For example, Mary has the following capability lists: (file1, read) and (file2, write), i.e., Mary has permissions to read file1 and to write into file2.

### 2.2. Mandatory Access Control

Like DAC, the U.S. DoD also defined the Mandatory Access Control (MAC) [56], as a means of restricting access to objects based on the sensitivity of the information contained in the objects and the formal authorization of subjects to access information of such sensitivity.

The following are the basic building blocks of a MAC system: Security Label and MAC Policy.

- Security Label: The security label can be seen as an access control mechanism that usually controls access to the objects and the users cannot alter the access permissions.
- MAC Policy: The MAC policy is the type of non-discretionary or mandatory security policy, where the individual owners do not have any choice to specify who can access what objects through access control policies.

Different security labels are associated with a MAC system. Unlike the traditional access control list, the MAC systems are the other forms of traditional security labels, such as role-based access control and attribute-based access control. In the following sections, we discuss both types of traditional MAC systems.

### 2.3. Role-Based Access Control

Among the access control mechanisms exist in the literature, the Role-Based Access Control (RBAC) mechanism [58] is a powerful and policy-based security solution for enforcing access control to information and data resources. It is mainly called a fundamental access control mechanism with the embodiment of the user-role and role-permission associations. RBAC has been widely accepted by different scientific and industrial communities to protect information and data resources due to its suitability and simplicity in administration when faced with a large number of users and permissions to data along with an overwhelming flow of data.

The following are the basic building blocks of the RBAC system: User, Role, Permission, User-Role Assignment Policy and Role-Permission Assignment Policy.

- User: In the RBAC model, subjects are typically represented by users, who are the human beings.
- Role: Users are assigned to roles based on their credentials or job functions in the organizations along with different sessions.
- Permission: Permissions (i.e., resource access permissions) are assigned to roles based on the authorities and responsibilities conferred on the users assigned to these roles.
- User-Role Assignment Policy: The user and role-specific attributes are used to specify user-role assignment policies. Users can create active sessions to activate roles and users’ assignment in roles can be revoked after the associated sessions.
- Role-Permission Assignment Policy: The role and permission-specific attributes are used to specify role-permission assignment policies. Users acquire resource access permissions by being members of roles.

The traditional RBAC system [58] is also extended to support different environments, incorporating the spatial, temporal and other conditions [16,18] into the RBAC policies. In accordance with the embodiment of the user-role and role-permission mappings, the traditional RBAC model [58] and spatial and temporal RBAC models [15,17,18] have been widely accepted by different scientific communities due to their flexibility and simplicity in administration when faced with a large number of users and a large amount of data.

### 2.4. Attribute-Based Access Control

The Attribute-Based Access Control (ABAC) [59] approach has also received considerable attention and popularity in the IT industry. ABAC grants access to resources and services based on the attributes of relevant entities. In particular, the ABAC permissions are associated with attributes, but not with roles. Attributes can be identified based on the domain in which ABAC is applied. The subject-specific attributes are very important in ABAC system as they specify the properties of the users who attempt to access the resources, however, it has another type of attributes. The ABAC policies define which constraints need to be satisfied based on the attributes of entities (e.g., the user is located at the emergency room), and eventually determine how accesses to resources are controlled.

The following three categories of attributes are included in the ABAC system, including the attribute-based policy as building blocks.

- Subject-Specific Attribute: Subject-specific attributes describe the users who attempt to access, such as the age, role, and job title.
- Object-Specific Attribute: Object-specific attributes describe the data or resources being access by the users, such as the granularity or type (the daily medical records, the medical history), the sensitivity (the critical or emergency medical record, the private medical records), the location.
- Action: Action-specific attributes describe the action (on resources) being attempted, such as read, write, update, delete.
- Attribute-Based Policy: In ABAC, the access control policies are used to limiting access to certain resources, based on the attributes, e.g., “user.age = 30”.

### 2.5. Discussion

Though in this survey, the Role-Based Access Control (RBAC) mechanism has been studied, the Attribute-Based Access Control (ABAC) concept provides us useful insight for modelling a wide range of dynamic attributes (i.e., contextual conditions). One of the biggest limitations of the traditional access control models, such as DAC and MAC, is that they manage access privileges based on individuals, instead of groups or roles of individuals. Whenever the numbers of users and resources are high, the amount of authorization policies can become extremely large, which complicates the administration tasks. This brings a high complexity of security administration and significant cost of managing large-scale systems.

Unlike DAC and MAC which are identity-based access control models, the RBAC models are based on the roles of users. In these models, the user and permission assignments are static without taking into account the dynamic attributes (i.e., context information), such as the location and request time. For instance, a nurse who is not localized in the hospital should not have the right to access the medical records of patients. Moreover, this access is allowed only during her ward shift time. However, the ABAC models are the rule-based approach to access control. These models are easy to set up but complex to manage in the large-scale systems, because of the huge numbers of attributes.

These traditional access control mechanisms are limited in order to provide the “granted” or “denied” access control decision to the users by satisfying the dynamically changing contextual conditions. In addition, these traditional systems do not provide adequate functionalities to access required data from distributed cloud and fog environments.

## 3. The Context-Aware Access Control

This section includes the background of pervasive context-aware computing and access control domains.

### 3.1. Context Information in Pervasive Computing Domain

In today’s dynamic era of pervasive and ubiquitous computing, the dynamically changing contextual conditions (that we simply mean “context”), such as the temporal, spatial and interpersonal relationship information, have been playing a major role to maintain the privacy and security requirements of the associated stakeholders. In the following sections, we discuss the different types of context information along with a taxonomy and different definitions of context.

Many researchers have attempted to define the concept of context. The generalized definition of context given by Dey, who defines context as any information about the general entities: person, place or object [60,61]. This definition does not specify the different types of entities specific to access control or the wide range of context information characterizing these entities (e.g., the relationships between relevant entities). Dey’s definition of context further has been specialized to specifically cover context-aware access control applications [62], in which the context means any information about the access control-specific entities: user (including resource owner), data/resource or environment.

Context-awareness is an important aspect of the dynamically changing environments, where users typically access resources (information, services, etc.) in an anywhere-anytime fashion. Access control in such environments needs to be dynamic and context-aware, taking into account the relevant contextual information. By leveraging the dynamically changing contextual information, we can achieve context-specific control over access to information resources and services, better satisfying the security and privacy requirements of the stakeholders. Therefore, there is an increasing need for new access control approaches to link their decision-making abilities with context-awareness in recent years.

The dynamically changing conditions (e.g., the physical location) constitute the contextual information. Based on the Context-Aware Access Control (CAAC) research [62], the context information can be categorized as the user, resource, and their environment-specific conditions. There is always an association between a system (a system constitutes by the users and data resources) and its environment, and the system should always adapt to its ever-changing dynamic situational environment. This situation constitutes the CAAC systems and the method is called “Context-Awareness” for such systems.

### 3.2. Context Information in Access Control Domain

We first present different dynamic conditions (the terms contextual conditions or contexts or contextual information have been used interchangeably throughout in this paper) that usually are associated with relevant contextual entities. We then present a taxonomy of contextual conditions according to the access control-specific contextual entities, which can be a user (including the resource owner), resource, or their surrounding environments.

Table 2 demonstrates different contextual entities, staring from the general pervasive domain to access control domain.

**Figure 2:**
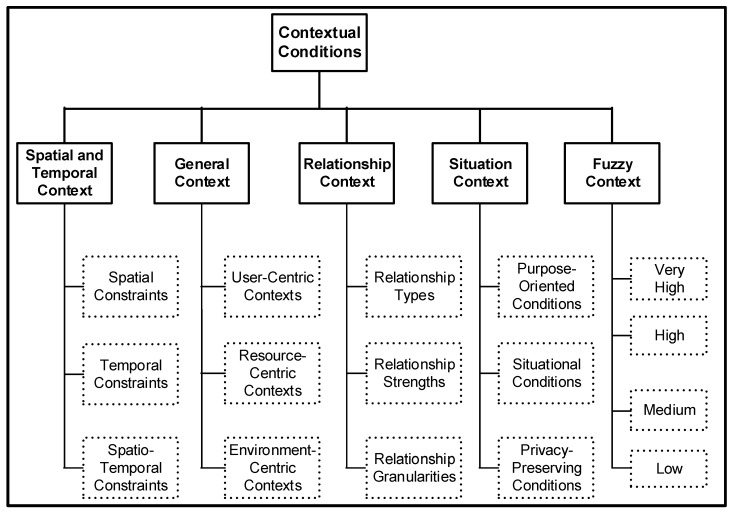
A taxonomy of contextual conditions.

Our criteria for defining the taxonomy is based on the key issues in context-sensitive role-based access control (e.g., spatial RBAC [18], temporal RBAC [15]) and how they have been tackled in the existing RBAC literature. As illustrated in Figure 2, the contextual condition can be grouped as spatial, temporal, general, relationship, situation and fuzzy contextual conditions. The categories of contextual condition at the top tier of the taxonomy (see Figure 2) are the main areas, which are expanded further in order to cover different context-aware RBAC (simply CAAC) research areas. In Section 4, we further discuss and elaborate all the different types of contexts that are associated with different access control mechanisms.

### 3.3. Context-Aware Access Control

The context-sensitive access control system can be seen as Context-Aware Access Control (CAAC) [25], and in CAAC, the access permissions are associated with both attributes and roles, where a context can be seen as an attribute. A set of contexts or contextual conditions are determined according to the domain in which CAAC is applied.

According to CAAC research [19,62], there are three types of contextual conditions (or contexts), such as user, resource, and their environment-centric information. Table 3 shows the conceptual background of the context information. Contexts can be grouped into the following three categories.

- User-Centric Context: User-centric contexts are the information about representing users. A user can be the resource requester, the resource owner or any other environmental person.
- Resource-Centric Contex: Resource-centric contexts are the information about representing data or information resources.
- Environment-Centric Context: Environment-centric contexts are the information about representing the surrounding environment between user and resource, such as the location from where the access request has been originated.

Comparing to RBAC system, the following are the basic building blocks of any CAAC system [25]: user, role, permission, context, context-aware user-role assignment policy, and context-aware role-permission assignment policy.

- User: Users are human-beings interacting with a computing system, whose access requests are being controlled.
- Role: Roles reflect users’ job functions within the organizations (e.g., in the healthcare domain).
- Permission: Permissions are the approvals to perform certain operations on resources, by the users who initiate access requests. The resources are the objects protected by access control that represent the data/information container (e.g., the patients’ medical records). The operations are the actions that can be executed on the resources, for instance, read operation of the patients’ medical records.
- Context: Contexts characterize the situation of entities, such as the users, resources or their environments, e.g., the physical location and the interpersonal relationship between user and resource owner. The expressions are used to express the dynamic contextual conditions (using relevant context and situation information) in order to specify the user-role and role-permission assignment policies.
- Context-aware user-role assignment policy: Context-aware user-role assignment policies are the many-to-many mapping between a set of users and roles, when a set of dynamic contextual conditions are satisfied.
- Context-aware role-permission assignment policy: Context-aware role-permission assignment policies are the many-to-many mapping between a set of roles and permissions when a set of dynamic contextual conditions are sanitised.

**Figure 3:**
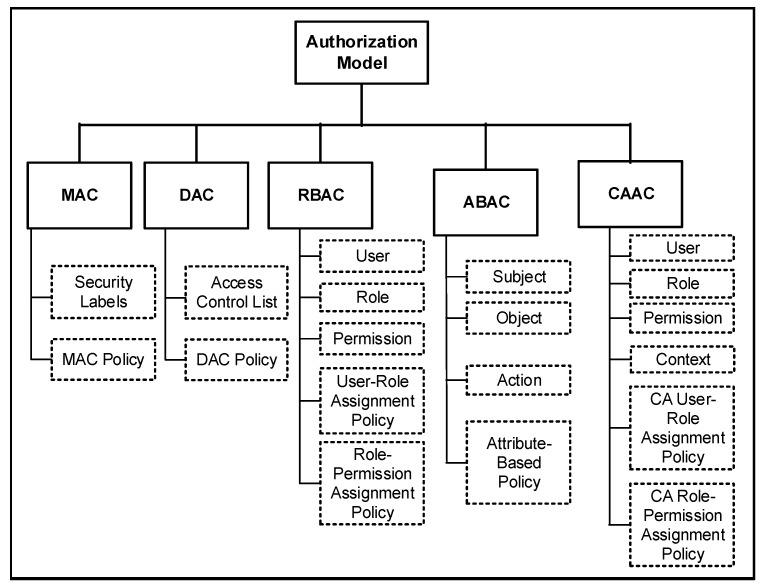
A taxonomy of authorization models.

The building blocks of the ABAC, RBAC, DAC and MAC systems are already discussed in Section 2. Figure 3 demonstrates a taxonomy of authorization models in different access control systems, including other key building blocks of those systems. Different security labels are associated with MAC-based access control systems, whereas the access control list is associated with the DAC-based systems.

Since computing technologies have become increasingly pervasive in our everyday lives, the security and privacy protection mechanisms are moving from fixed desktop environments to dynamic environments, such as ubiquitous and cloud-based environments. The different access control technologies have historically been applied to appropriately control data and information resources in such environments. Different contextual conditions, such as user profile information, social relationship information and so on, are incorporated into the above-mentioned basic, spatial and temporal RBAC approaches to support these dynamic environments.

Among them, the Context-Aware Access Control (CAAC) mechanisms [13,14,22,25] have been historically applied in today’s dynamic and interconnected environments. These CAAC approaches usually consider the different types of contextual conditions for making access control decisions. Deriving relevant contextual conditions from relevant data sources and different environments explicitly for such access control mechanisms is an important research direction over the last few decades. Similarly, in today’s cloud-based interconnected world, we need a new generation of access control mechanism to support data access from multiple, distributed sources.

### 3.4. Discussion

Some of the existing Context-Aware Access Control (CAAC) models which are RBAC-extended models, consider the location (i.e., spatial constraints), time (i.e., temporal constraints), or both (i.e., spatio-temporal constraints) as context information for defining and enforcing access control policies. Like the traditional RBAC models described in the earlier section, these contextual RBAC-extended models have the same limitations when used in pervasive context-aware environments. There is a need for incorporating a general context model into the policy model for providing dynamic access control decisions.

In the field of pervasive context-aware computing, the context information represents the general context entities such as person, place and object, without considering the access control-specific context entities and information. The existing access control approaches and their associated context models are not adequate to capture and reason about all the different types of contextual information such as the information characterizing the relationships between entities. On the other hand, the specific context modelling approaches (e.g., [64,65,66]) do not provide direct context modelling support for concepts related to access control. Therefore, there is a need for a comprehensive and extensible access control-specific context model for modelling a wide range of dynamic contextual conditions. In addition, there is a need for inferring high-level (implicit) context information from the other available context information.

With the advancement of IoTs and cloud technologies, users are currently moving their data from centralized environments to distributed cloud networks, however, there is still a lack of an appropriate access control mechanism to control and access data from distributed environments. Nowadays, social networking platforms (such as Facebook, Instagram, etc.) have been offering many online services to different communities utilizing cloud computing infrastructure. However, there is still a lack of considering the privacy and service agreements of such social service providers. The existing CAAC mechanisms do not support an integrated or resultant view of data resources from multiple cloud environments. Therefore, there is a strong need to develop new context-sensitive access control solutions, utilizing the benefits of a single set of policies in order to access different data resources from distributed cloud and fog environments with low computational overheads.

## 4. The Context-Aware Access Control Approaches and Frameworks for Centralized Networks

In this section, we survey the existing CAAC approaches and frameworks for centralized network environments, by following our introduced taxonomy of context information. We also survey the relevant contextual conditions that have been incorporated into the security policies in the existing access control mechanisms.

### 4.1. The RBAC Approaches with Spatial and Temporal Contexts

Bertino et al. [15] have proposed the temporal RBAC (TRBAC) approach, which extends the traditional RBAC approach [58] in order to support temporal constraints on enabling/disabling roles. They have defined a concept, named Role Enabling Base (REB), to describe temporal constraints on the enabling of roles. The REB is composed of Periodic Events (PE) and Role Triggers (RT). Periodic events have the form <I, P, p:E>, where ‘I’ is a time interval, ‘P’ is a period expression, and ‘p:E’ is a prioritized event expression. For example, the periodic events and role triggers in the REB state that the doctor-on-night-duty role must be enabled during the night. Later, Joshi et al. [17] have extended the TRBAC approach proposed in [15]. They have proposed a generalized temporal role-based access control (GTRBAC) model [17] that allows specification of a comprehensive set of time-based access control policies, incorporating the temporal constraints in both user-role and role-permission assignment policies.

A well-known spatial access control approach is GEO-RBAC [67], which extends the traditional RBAC approach [58] with the concept of spatial role. A spatial role represents a geographically bounded organizational function. The boundary specifies the spatial extent to which the user is located and enabled to play such a role (spatial role). In GEO-RBAC, access control to resources and services is based on the location of the users and their assigned roles. Later, Zhang et al. [16] have proposed a location-aware RBAC approach named LRBAC. In the LRBAC approach, the concepts of spatial role and effective role (like RBAC roles) are introduced. LRBAC models the user locations and geographically bounded roles. These roles are automatically activated/deactivated by the locations of the users. In LRBAC, both the activated roles of the users and their locations are taken into account in order to evaluate the access control policies.

Chandran et al. [68] have extended the GTRBAC approach [17], and proposed a location and time-based RBAC approach, named LoT-RBAC. LoT-RBAC addresses access control requirements of dynamic environments in order to provide location and time-based access control. LoT-RBAC has three main RBAC entities, users, roles and permissions, and considers temporal and spatial contextual information. LoT-RBAC adopts and extends the concepts of role activation and role assignment from the basic RBAC approach, according to temporal and spatial contextual information. In particular, a role is activated by a user from the location ‘l’ at time ‘t’, if the location and temporal information of a user associated with the role activation are satisfied. Bhatti et al. [69] have proposed a context-aware access control approach, named X-GTRBAC. The X-GTRBAC approach extends the generalized temporal role-based access control (GTRBAC) approach [17]. GTRBAC provides a mechanism to express a diverse set of fine-grained temporal constraints on user-role and role-permission assignments in order to meet the dynamic access control requirements of an enterprise. X-GTRBAC adopts the temporal-aware user-role and role-permission assignment policies from the GTRBAC approach, and considers spatial context information in these assignments.

The above-mentioned extended RBAC approaches (i.e., spatial, temporal and spatio-temporal RBAC mechanisms) take into account the temporal and location information when enforcing access control policies. However, they do not provide adequate methodological and implementation supports to model a unified set of access control policies with respect to accessing data from multiple sources with low overheads. Other than the traditional RBAC and extended RBAC mechanisms, we have a successful history of developing a family of context-aware RBAC mechanisms. As examples, in the following sections, we have considered the general context information [6], the relationship context information [20] and the situational context information [10], the dynamic contextual role information [8], and the fuzzy context information [7]. Recently, a new secure version of an RBAC approach has also been introduced [70].

### 4.2. The RBAC Approaches with User, Resource, and Environment-Centric Contexts

Al-Kahtani and Sandhu [71] have introduced an extended RBAC approach through rules, called rule-based RBAC (RB-RBAC). In this approach, users are dynamically assigned to roles based on a finite set of assignment rules derived from the security policy. These rules take into consideration the attributes of users as contextual information. Similar to RB-RBAC, Kern and Walhorn [72] have adopted RBAC approach and proposed a rule-based provisioning system for the RBAC approach based on a limited set of user attributes as contextual information. These approaches have the limitation of considering only the user-centric contextual information as policy constraints.

Later, Zheng et al. [73] have proposed a dynamic role-based access control (DRBAC) approach, which incorporates the required credentials of users as contextual information when making user-role and role-permission assignments. The DRBAC approach extends the basic RBAC approach and it dynamically grants and adapts permission to users according to users’ contexts.

In 2012, Kayes et al. have proposed a basic Context-Aware role-based Access Control (CAAC) framework for dynamic centralized network environments that are based on semantic technologies [62], and later the same authors extended the initial CAAC policy model by incorporating a wide range of contextual conditions [19]. Recently, Kayes et al. [6,25] have introduced a context-aware RBAC policy model for data and information resources. The dynamically changing contextual conditions (or contexts) constitute what is widely known as contextual information [62]. According to context-sensitive access control research (e.g., [25]), there are three groups of contextual conditions, such as user, resource, and their surrounding environment-centric information (e.g., patients’ profiles, users’ locations, users’ request times). For instance, the interpersonal relationship between two users can be seen as user-centric contextual information.

### 4.3. The RBAC Approaches with Relationship Contexts

Zhang et al. [74] have proposed an approach named relation-based access control (RelBAC), in which the access permissions are formalized as binary relations between subjects and objects. They consider the subject, object and permission as the compulsory access control components. Fong et al. [75] have presented a relationship-based access control approach named ReBAC. They consider the relationships between individual users (e.g., professional association) in the access control policies. Both RelBAC and ReBAC approaches consider the expression of access control policies in terms of the relationship granularity (e.g., friend, friend-of-friend, friend-of-friend-of-friend).

Recently, Kayes et al. [20] have introduced a relationship-aware RBAC approach, incorporating the relationship context information such as the different granularity levels of relationship, the relationship types, the relationship strengths into the access control policies. In particular, the authors have proposed a relationship ontology to dynamically identify much richer forms of relationships among relevant entities with different granularity levels and strengths of that relationship, and a policy ontology to provide relationship-aware access to information resources based on the inferred relationship context information.

While these existing CAAC mechanisms consider a broader range of contextual dimensions and provide useful insight for modelling context-aware role-based access control concepts, they are still limited in considering a diverse range of environmental factors or context information (e.g., different types of relationships) along with context inference mechanism to reason about some richer context information from the available information.

### 4.4. The RBAC Approaches with Situational Contexts

In different context-aware access control approaches, the traditional RBAC model [58] is also extended with considering different situational conditions, which can be seen as situation-aware access control mechanisms.

In the literature, many researchers have attempted to define the concept of the situation. As defined by Endsley [76], the situation comprises the perception of the elements in the environment within a volume of time and space, the comprehension of their meaning, and the projection of their status in the near future. This definition has been widely accepted by the information technology community. In the pervasive context-aware computing literature, Wang et al. [64] have described user-specific situation-awareness only concentrating on the state of the user.

Kim and Lim [77] have proposed the Situation-Aware RBAC (SA-RBAC) approach, which has extended the basic RBAC approach and grants permissions to users based on the situational conditions. The SA-RBAC approach is used to deal with the situation information by considering the combination of the required credentials of context entities. SA-RBAC grants users to roles and roles to permissions based on the situation information. Later, Garcia-Morchon and Wehrle [78] proposed a two-layer modular context-aware access control approach for medical sensor networks comprising a data layer and an engine layer. The data layer comprises all the information (including context information such as location and time) that is required for access control decisions. The engine layer manages the access control decisions in critical, emergency and normal situations using this context information and access control policies. This access control approach adopts the traditional RBAC approach [58] and incorporates the situation information into the access control process. Yau and Huang [79] have presented a situation-aware ontology in service-based system to ensure the satisfaction of user’s quality-of-service (QoS) requirements in dynamically changing environments. They consider the situation as a set of context attributes of users and other entities involved in computing.

In the literature, different researchers consider privacy-preserving constraints as situation conditions. Yau and others [80] have defined the situation as a set of context attributes of users, systems and environments over a period of time affecting future system behaviour. Later, Yau and Liu [81] have presented a Situation-Aware Access Control (SA-AC) approach for privacy-preserving service matchmaking. The SA-AC approach incorporates privacy-preserving constraints into basic RBAC approach, such that the dynamic states of service providers, requesters and environments are considered, in order to safeguard access control decisions. The SA-AC model includes these constraints into both user-role and role-permission assignments as situations. SA-AC provides an XML-based access control language for specifying flexible access control policies.

The above mentioned situation-aware approaches consider the situation as the specific combinations of context information (e.g., the states of the relevant entities). In dynamic and context-aware environments, however, the states of the relevant relationships between different context entities is also an important consideration in access control decision making. Furthermore, the purpose or user’s intention in accessing resources and services is another important aspect that needs to be considered. Therefore, a purpose-oriented situation model to identify relevant situation information is proposed by Kayes et al. [10,21]. In this model, an ontology-based situation-aware RBAC approach has been proposed, incorporating the purpose-oriented situation information (e.g., accessing data for normal/emergency treatment purpose, research purpose) into the policies.

The traditional RBAC model [58] has considered the static role for managing access control decisions, for instance, the daily medical records of a patient that has been assigned to a hospital Nurse role and Mary, who is a hospital nurse, can access such records. The limited scale contextual roles has been considered earlier, for example, [67] have extended the RBAC static role and proposed a spatial role that represents a geographically bounded organizational function. Joshi et al. [17] have also extended the RBAC static role and proposed a temporal role, for example, the ‘doctor-on-day-duty’ role must be enabled during the day.

Recently, Kayes et al. [8] have introduced a new CAAC approach for the specification and activation of dynamic contextual role at runtime, where the dynamic contextual role instead of static role is considered as situational constraint, aiming to increase resource privacy and confidentiality. The basis of this approach is RBAC role hierarchy with the extension of a contextual role hierarchy. A contextual role represents the users’ state based on the dynamic changing contextual conditions. In this approach, a policy specifies a particular operation to be performed on an object (i.e., resource access permission) based on activating the relevant contextual role and it is represented as a tuple in the form of <user, contextual role, resource>.

In general, there is still a lack of an extensible context and/or situation model that can capture the wider range of context information, a reasoning technique to infer richer context or situation information, and a context-aware policy model to incorporate these different types of contextual/situational information to provide context-specific access to data and information resources.

### 4.5. The RBAC Approaches with Fuzzy Contexts

Jones et al. [82] have extended the widely accepted definition of the situation (by Endsley [76]) and proposed a fuzzy cognitive mapping techniques to model situation awareness for army infantry platoon leaders.

Almenárez et al. [83] have proposed a trust-based access control approach based on the trust values of high, medium and low [84], allowing only authorized users to access sensitive data (and information resources) that are usually confidential. Takabi et al. [85] have proposed a trust-based RBAC approach to online services, using different fuzzy relations to compute trust values from the relevant attributes (e.g., behavioural, personal). Martínez-García et al. [86] have proposed a fuzzy RBAC approach to deal with access control-specific imprecise information through fuzzy relations.

Recently, a fuzzy context information system along with a CAAC policy model for access control decision making has been introduced [7,63], utilizing the basic fuzzy model [87] to derive the fuzzy contextual conditions. Kayes et al. have introduced a policy framework for context-sensitive access control where fuzzy and other contextual conditions are incorporated to make access control decisions [25].

The contextual conditions are obtained mostly by exploiting the classical crisp sets and have been incorporated into the access control policies [22]. However, there are some conditions that cannot be obtained directly from crisp sets, the range of the degree of membership values is either 0 or 1. As such, Kayes et al. [7] exploited the fuzzy sets in terms of an appropriate way to derive such fuzzy context information by proposing a fuzzy context model. For example, a patient’s current health condition is derived “95% critical”, i.e., criticality level is “very high”, from the low-level data such as pulse rate and body temperature. The location and request time of a health professional can be obtained directly from the associated crisp sets (e.g., the location is in the emergency room or not, i.e., the crisp set = {1, 0}). The health status cannot be obtained directly, however, it can be obtained from the low-level contextual data (e.g., the health status is “5% normal” with “criticality level 95% or 0.95”). Very recently, Kayes et al. [63] have extended the earlier fuzzy model [7] and proposed a new context-aware access control approach for cloud-based data resources.

These existing fuzzy context models only consider the limited contextual or environmental factors, such as the degree of membership values like low, medium, high and very high. However, these CAAC mechanisms and their associated context models have similar drawbacks in capturing the specific types of fuzzy context information and in inferring the richer context information in a systematic manner.

### 4.6. Discussion on Access Control Mechanisms for Centralized Networks

Table 4 presents the different access control mechanisms for centralized networks. Focusing on the survey of different context-sensitive access control approaches and frameworks in centralized environments, we have the following observations.

The different access control models and approaches have been applied in different environments. Among them, the traditional Role-Based Access Control (RBAC) approach [58] is the fundamental security model to protect data and information resources. The basic RBAC approach has become the most widely used access control model. It typically evaluates access permission through roles assigned to users.

On the other hand, the dynamic attributes are incorporated with RBAC approach to grant access permissions to resources based on the attributes of relevant entities and the users’ roles. As such, the spatial, temporal and other context-aware RBAC approaches have been applied in the centralized environments. More recently, some context-aware access control approaches have been introduced and they extend the basic RBAC approach with specific types of dynamic contextual information. However, these approaches and their associated context models are not adequate to capture and reason about all the different types of contextual information such as the information characterizing the relationships between entities.

Existing situation-aware access control approaches grant access to resources depending on the specific combinations of certain contextual information. However, they are still limited in considering the richer context information and an appropriate inference mechanism to derive high-level information (e.g., fuzzy context information). Furthermore, the existing access control policy models do not have adequate functionalities to incorporate diverse context and situation information into user-role and role-permission assignments for dynamic access control decision making.

Let us consider some practical scenarios in order to discuss the suitability of the access control techniques.

- John, who is a paramedic, can provide emergency treatments to save a patient’s life from a critical accident situation, by accessing the patient’s medical records, previous historical data and private health records. However, he needs to satisfy the associated contextual conditions (e.g., ‘co-located’ with the patient at the scene of an accident when the patient’s health situation is ‘critical’).
- A patient’s current health condition is derived as “98% critical”, i.e., criticality level is “very high”, from the low-level contextual facts such as heartbeat and body temperature. Due to the dynamic nature of computing technologies, there is still a growing need to exploit further contextual conditions derived from information provided through IoTs and relevant environments, in order to control context-sensitive access to data and information resources at different granularity levels.
- A hospital doctor is allowed to activate an “emergency doctor” role at the emergency department of the hospital when the patient’s health condition is “highly critical”.

The above-mentioned access control approaches are applicable in the centralized environments, ranging from database management systems. However, these context-sensitive RBAC models are not adequate to overcome the limitations and challenges of managing access and privacy control to the distributed environments, such as in today’s cloud and fog networks. Overall, the traditional RBAC frameworks and the extended-RBAC frameworks with different contextual conditions (e.g., spatial, temporal, situational information) are limited in addressing issues like extensibility and reasoning over security (access control) policies. On the other hand, most of the classical CAAC frameworks do not address the issue of dynamic nature of distributed networks.

## 5. The Access Control Approaches and Frameworks for Decentralized Cloud and Fog Networks

In this section, we survey different context-sensitive access control and data sharing mechanisms that can be applicable in decentralized (or distributed) network environments (e.g., cloud and fog levels). We also identify the associated research challenges.

### 5.1. The CAAC Approaches for Accessing Data from Edge, IoT and Cloud Networks

Over the last decade, data storage in the cloud has become an important trend. Storage, computing and network management has been shifted to centralized data centres. Cloud offers infrastructure, platforms and software as services (IaaS, PaaS, SaaS). Cloud computing is attractive as the business owner need not to invest in infrastructure and can rent resources from cloud service providers as per their needs and usage. In addition to this, several other technical benefits include resource optimization, optimization of hardware and software, energy efficiency, elasticity and flexibility [88]. However, a major problem in computer security has always been access control, a selective restriction of any data or resource. Protecting resources just from a system perspective is not enough anymore, the access control models must be able to suit the dynamic environment and adjust distributed permissions accordingly.

Lately, more and more researchers have realized the value of context in authorization. Many context-sensitive access control models such as GRBAC [89], X-GRBAC [69], GTRBAC [17], SC-RBAC [90], GEO-RBAC [18] have been proposed. In the cloud, the relation between users and resources is dynamic, so users and service providers might not be in the same security domain and hence identity-based securities such as Discretionary access control (DAC) or Mandatory access control (MAC) cannot be used in an open cloud computing environment. Zhou et al. [91] proposed a new Context-Aware Access Control for cloud computing (CAACM) by considering platform trust level, spatial state and temporal state as context. The particular model determines mechanisms of authorization from cloud management role to objects allowing dynamic activation of role permission by identifying cloud management role with context. The model guarantees multi-level security and achieves fine-grained access control by including the hierarchy of managerial roles and associating it with the context. CAACM is appropriate for systems on cloud computing including outsource-oriented systems.

Though there are existing access control models that can be applied in the cloud networks, accessing data from multiple sources (e.g., multiple databases, distributed clouds and so on) has increasingly become challenging nowadays due to the multiple natures of IoT data sources and an overwhelming flow and storage of data in the cloud. In the literature, several cloud and fog-based access control approaches have been proposed recently to access data from multiple sources, incorporating different contextual conditions into the access control policies.

Kayes et al. [22] have introduced a new generation of context-aware access control framework to access data from multiple cloud databases that can be applied to the distributed network domain. Two building blocks of the framework are a global data ontology and its mapping model in order to utilize the benefits of a unified set of access control policies and overcome the computational overhead issues accordingly. This fog-oriented CAAC approach [22] can be applied to access data from multiple databases utilizing a unified set of access control policies. However, this access control approach is not adequate to protect personally identifiable information obtained from multiple data sources. Very recently, Kayes et al. [23] have extended the initial fog-oriented CAAC framework [22] and introduced a new Fog-Based CAAC (FB-CAAC) approach to collate data from multiple sources and provide an integrated data view to the users, by maintaining the privacy and security requirements of the stakeholders.

A number of surveys have been undertaken to assess the privacy and security challenges in fog computing and the internet of things [92,93,94]. In these surveys, the authors have focused on several issues: (i) the integration of IoT and cloud computing platforms considering the limited computational power and storage capacity of the IoT platform, (ii) providing efficient and secure services for end-users through fog computing like data processing and storage locally at IoT devices, and (iii) other security and privacy concerns of fog computing as the existing privacy and security solutions of cloud computing cannot be directly applied to the fog network.

Pierleoni et al. [95] have presented an in-depth analysis of architectures highlighting different security approaches to cloud-IoT platforms. Jiang et al. [96] have proposed a trust-based data collection framework for edge networks by identifying the trust degree of sensor nodes. Liu et al. [97] have introduced an adaptive data-centric security approach to collect big data from multiple sensor nodes. Huang et al. [98] have proposed an effective service-oriented architecture for IoT data, by reducing traffic load through service aggregation at the network layer. However, the existing architectures and frameworks are not adequate for accessing data from multiple edge/sensor and cloud-based networks.

### 5.2. The Privacy-Preserving Protocols and Mechanisms for Distributed Cloud Databases

The recent progress in cloud computing environments in conjunction with the Internet of Things (IoTs) has provided the opportunity to gain a better life at present. However, such IoT-enabled infrastructures and applications are posing new challenges in preserving the privacy of the users and securing data from unauthorized parties. In the literature, several privacy-preserving protocols and mechanisms have been proposed for protecting confidential and sensitive data in the cloud networks.

Saha et al. [99] have introduced an IoT-based healthcare framework, employing fog layers to providing fast response time and low latency. The proposed framework deals with electronic medical records and preserves the privacy issues. The authors have conducted experiments with respect to response time and delay and have compared their framework with recent works. The results show that the proposed framework is efficient in providing privacy along with standard network parameters.

Siow et al. [100] have built a personal datastore to process, publish and store IoT time-series data in a smart home application, including historical and dynamic streaming data. In addition, they have demonstrated a real-time dashboard that can visualise historical IoT data. We can utilise this datastore to process the time-series IoT data from multiple sources. However, this datastore is not adequate to integrate IoT time-series data with other contextual data (e.g., patients’ locations). How to deal with legal and cyber issues of the IoT data that are coming from multiple sources is still a research issue.

Agrawal and Srikant [101] have addressed the concrete problem of building decision tree classifier while respecting user’s privacy concerns. They perturb the data using Uniform and Gaussian methods and reconstruction is done in a global, byclass and local way from which their results say that byclass and local mathods are effective algorithms. The proposed method can preserve the privacy of individual records as it reconstructs just the distribution and not individual records.

Doganay et al. [102] have used additive secret sharing instead of costly Public Key Encryption as a cryptographic primitive to implement secure multiparty computation protocol. Advantage of using secret sharing instead of Public Key Encryption is that it does not suffer from bit expansion. They also try to decrease the communication and computation costs. Unlike the previous paper [102], Erkin et al. [103] have employed cryptographic tools with data packing to reduce computation and communication cost.

Oliveira and Zaiane [104] have introduced a family of geometric transformation methods that distort confidential numerical attributes in order to meet privacy requirements in cluster analysis. This paper was a building block towards privacy-preserving data clustering. Badsha et al. [105,106] have proposed a privacy-preserving protocol for web service recommendation with negligible loss of accuracy of QoS values by leveraging different encryption techniques.

Badsha et al. [107] have proposed a new privacy-preserving user filtering protocol based on the locations where the users, who are not within a given region, can be eliminated. Recently, Badsha et al. [108,109] have proposed a privacy-preserving collaborative cyber threat information sharing framework leveraging Homomorphic Encryption and content-centric network leveraging public key attribute-based encryption respectively.

### 5.3. The Privacy-Preserving Mechanisms for Cloud Service Providers

Wang et al. [110] have introduced an approach to integrity checking by the third party so that the unauthorized users would not learn any knowledge about the data content stored in the cloud servers during the auditing process. However, this research does not utilize cryptography method effectively to securely integrate data in cloud computing environment.

Alabdulatif et al. [111] have proposed a framework and applied a lightweight encryption mechanism on sensed data prior to analysis on the cloud. The framework preserves data privacy without affecting the accuracy of the anomaly detection process. However, there is a lot of communication overheads involved between trusted private servers and cloud infrastructures. Recently, Alabdulatif et al. [112] have worked towards reducing the high communication and computational overheads involved in the cloud by using a distributed approach that improves performance.

Van and Juels [113] have listed the limitations of trusted platform modules and cryptography alone in meeting the challenges of privacy in the cloud. They have suggested the use of a stateful multi-client model that leads to the distribution of trust along with cryptography, which helps to verify specific security requirements of cloud deployment.

Malina and Hajny [114] have proposed a solution to offer anonymous access, unlinkability and the confidentiality of transmitted data over the cloud to ensure anonymous authentication between cloud service providers and clients. Xu and Joshi [115] have proposed an integrated user-centric privacy-preserving attribute-based access control approach to protect the security and privacy of users’ data stored by a cloud service provider using revocable ciphertext attribute-based encryption scheme.

### 5.4. The Policy-Aware Deployment and Management of Cloud Applications

The cloud users first develop their cloud applications, and then deploy and manage the developed applications across one or more cloud providers. The policies are used to define the security and privacy requirements that should be respected during the deployment and management of cloud applications. The cloud providers need to be able to enforce such policies.

Policy4TOSCA [116] supports the design, implementation, and application of the policies in the cloud application deployment models defined in the TOSCA (Topology and Orchestration Specification for Cloud Applications) standard. The policies are used to express the non-functional requirements such as cost and security, pertaining to deployment, management, and use of the cloud applications. For example, to achieve data protection, security policies can be used to instruct encrypting databases and restricting the psychical location of the cloud data centres to be used for hosting application components. An extension to Policy4TOSCA has been proposed by Breitenbücher et al. [117] for automated generation of the policy-aware provisioning and management plans (workflows).

Yussupov et al. [118,119] have proposed a policy-based deployment approach to model the security requirements used in a collaborative cloud application development environment. The deployment model can be exchanged among partners without violating modelled requirements. Security requirements cover the protection of data confidentiality in deployment models (encryption policies), and the verification of data integrity and authenticity of deployment models (signing policies). An extension to TOSCA was provided to attach the security policies to the deployment. Cryptographic Access Control (CAC) was employed to define and enforce the access rights (for the part of deployment models) for partners in the collaborative environment. The policies can also be used to model and automate the execution of application deployment tests to verify that the application behaviours, including security functionality [120].

Zimmermann et al. [121] have proposed a rule-based approach to enforce the data security and privacy requirements pertaining to the deployment of a distributed application across multiple data centres (i.e., cloud providers). The rules are specified to govern the provisioning process, for example, restricting the physical location where an application is allowed to be provisioned or restricting the selection of specific operating systems. Fischer et al. [122] have also used a rule-based approach to define and enforce the compliance requirements (e.g., regulation on storing of customer data) of an organization on the deployment models.

Kepes et al. [123] has introduced a framework that supports the deployment of a distributed application over heterogeneous environments such as clouds, private data centres, and IoT/Egde devices. They have proposed an agent-less, hierarchical deployment architecture to cope with the security mechanisms such as firewalls that may prevent proper access (e.g., inbound communication) for deployment, the restricted computing and storage capabilities of IoT devices, and dynamic locations of devices.

### 5.5. Discussion on Access Control Mechanisms for Decentralized Networks

Table 5 presents the different access control mechanisms for decentralized cloud and fog networks. Focusing on the survey of different context-sensitive access control frameworks and privacy-preserving access control protocols in the decentralized cloud and fog environments, we have the following observations.

The context-aware RBAC approaches have multiple advantages while applying in the traditional centralized networks, however, the policy models that are the cornerstone of those approaches are not applicable to access data and information resources from distributed cloud and fog networks, and for deploying, managing and protecting cloud-based applications.

In the literature, there are context-sensitive fog-based access control mechanisms that have been proposed very recently and applied in today’s dynamic and interconnected decentralized world. These mechanisms already have been engineered and accepted by different scientific communities due to their simplicity in administration and scalability (i.e., computational and processing overheads) in controlling data from multiple sources with a large number of users. These fog-based CAAC mechanisms can handle issues like accessing data from multiple cloud and fog networks and provide an integrated data view to the used by preserving privacy.

In addition, different cryptography security and privacy-preserving protocols have been introduced to handle issues like privacy protection in cloud-based applications, privacy preservation for cloud service providers and policy-aware management of cloud applications.

## 6. Towards a New Generation of CAAC Framework: Gains and Open Issues

In this section, we discuss several open research issues for safeguarding data and information resources using the dynamic CAAC mechanism. In particular, we highlight the challenges pertaining to existing CAAC solutions based on several real-world case studies that started to realize a new generation of access control framework for cloud-based data resources.

### 6.1. Future Research Directions and Real-World Case Studies

We identify several open research issues and challenges (along with the relevant case studies) that are not well addressed in the literature while accessing data from distributed cloud/IoT sources and safeguarding data against unauthorized parties.

1. Access management against identity thefts.
2. Safeguarding health records against data breaches.
3. Protecting banking customers against data breaches.
4. Security and privacy of the internet of things.

#### 6.1.1. Access Management against Identity Theft

OAuth [124] is a widely used Identity and Access Management (IAM) protocol that has been built on top of HTTP, however, the dynamism of the Internet that is intrinsic to the digital age has involved the production and consumption of a tremendous amount of user-generated data, especially via IoT (Internet of Things) platforms. We have been experiencing very poor consequences as such data surfaces are open for more attacks and data breaches. An IoT platform is mainly interconnected devices, sensors and actuators which are provided with unique identification numbers.

Identity theft is one of the burning issues is revolved around such a platform, which is still be causing problems in the era of today’s Internet, i.e., in the techno-social digital societies, like credit card or authentication token thefts and online frauds caused by data breaches. However, “how to protect people and their privacy, and build safer environments by looking at specific challenges that devices, sensors and actuators have, and look for new context-aware IAM mechanisms to address them” is still a research issue.

Let us consider an online banking example where an identity theft may cause significant psychological and economic damage to the banking customers. How identity theft happens in online banking and to seeking the right solution to stop it is a critical issue currently. It might be possible to detect identities by analyzing the metadata of users’ accounts. There might be some behavioural patterns between historical identity thefts cases, which also can be used in detecting future occurrences of identity thefts.

Let us consider another example, the two-factor authentication system that has been popularly implemented through Short Messaging Services (SMS) in Australian banking, is demonstrative of security vulnerabilities. Some preliminary findings in this direction has been presented in the earlier research [125]. In this study, Watters et al. [125] have examined 400 messages from websites that are used to receive SMS authentication tokens on behalf of banking customers, and found that 76.5% displayed the applications for which the message was intended and over three-quarters of the samples risked compromising their accounts. The research has been categorized mainly by two broad groups of people.

- Users who did not understand the consequences of such risks.
- Users who knew exactly as well as the consequences.

However, there is still a need for exploring the behaviour patterns of both groups using insights from theories of context-aware access control and machine learning.

#### 6.1.2. Safeguarding Health Records against Data Breaches

Data has been currently mounted up at a swift velocity and in real-time. We experience poor consequences as the data surfaces are open for more attacks and security breaches. We assert that such breaches are fundamentally failures of access control as most users are too busy or technically ill-equipped to understand access control policy expressions and decisions. Newspaper reports are filed on a near-daily basis in which the exploits of malicious actors (e.g., cybercriminals, terrorists) are described and explained to an immense online public.

On 1 January 2019, the Australian Digital Health Agency (ADHA) said that the number of data breaches has risen from time to time, from 35 incidents in 2018 to 42 data breaches so far in 2019, for example, incorrect health details entered into Australian My Health Record (MHR), and the wrong guardian assigned to MHR. Consequently, the MHR authority was forced to report many of these data breaches as “administrative errors” by the ADHA. Many Australians eventually opted out of the MHR scheme because of such ongoing security and privacy concerns.

On the one hand, like different health problems in ancient times, millions of Australians are currently despairing at this data breach war and they are worried about their privacy and health information, which might be sold and used by unauthorized third-parties, while on the other hand, it is necessary to disclose and give permission of their medical information to different health-related professionals towards a better treatment. There is still no clear guidance of the secondary use of health data where that is “in the public interest” [126].

How to prevent data breaches that involved in the MHR system is one of the burning issues currently. Security requirements elicitation is the main research issue revolved around herewith for measuring security vulnerabilities and data breaches in this MHR system. Currently, this system does not provide an explicit mechanism to engage in the decision-making processes, about who should have access to what data and when, that are involved in data breaches. However, how to obfuscate the attribution of responsibility and accountability for instances of security vulnerabilities is still an accompanying issue of remediation.

In short, one of the main challenges is who is responsible for what data, when, for how long, for what purposes and how can that responsibility be codified in a suitably transparent and ethical manner? How to estimate different tangible and intangible costs of data breaches is also an associated research challenge, considering the consequences of a data breach (e.g., loss of reputation).

#### 6.1.3. Protecting Banking Customers against Data Breaches

In the context of today’s interconnected societies, digitally networked technologies have increasingly bound their consumers and the confluence has created new opportunities for the attackers along with the associated vulnerabilities may open the rooms for data breaches. In recent time, the hackers have been successfully used a banking lookup service to retrieve and expose customers’ private details. Traditionally, the PayID system allows the bank account holders to reveal the details of their accounts, which is an online lookup through using the phone number or email address. According to the news of the Sydney Morning Herald, the private details of almost 100,000 Westpac customers have been exposed to a group of hackers in a recent data breach attack.

The traditional access and privacy control solutions have been proposed to control users’ access to data, for example, context-aware security mechanisms specific to access data, and privacy-aware mechanisms specific to manage privacy-sensitive data. The security and privacy policy models are the basic contributions of these mechanisms, which are the main cornerstones of today’s data-driven organizations, ranging from database management systems to operating systems. However, these existing solutions are not adequate to overcome the limitations and challenges of data breaches, such as problems related to the associated risks/attacks on baking customers. For example, though the Westpac’s money transfer platform takes the protection of customer data and privacy seriously, in the above-mentioned data breach attack, the platform allows the hackers to reveal the private details of account holders that also could be used for future frauds and secondary hacks. This is really a great challenge to identify threats and risks to current banking systems.

We have a successful history of developing a family of CAAC mechanisms to control data and information resources, where the capability to delegate CAAC access privileges is one of the essential components. Like the health records, one of the other important challenges is to control the secondary use of customers’ details and the associated frauds and hacks. Towards this goal, we may utilize the benefits of Artificial Intelligence (AI), data mining and machine learning techniques, which can deal with more fine-grained control of PayID lookup. We can identify the threats and risks to the banking systems while there are possible malware attacks and the associated data breaches. Our goal is to bring together the benefits of CAAC and AI, and do the following.

- Attack attribution.
- Attack forensics.
- Banking malware and fraud detection.
- False positive reduction for incident response.
- Threat intelligence and credential recovery in the dark web.
- Insider threat detection and behavioural analysis.

We can subsequently protect Australian banking customers from any unintended events, through utilizing the new era of CAAC and AI and detecting malicious/anomalous lookup.

#### 6.1.4. Security and Privacy of the Internet of Things

A world-wide network of interconnected objects called the Internet of Things (IoT) gained interest rapidly in the last several years. The reason for gaining popularity is, of course, its impact on every-day life of potential users. In the next future, IoT can play a leading role in both work and home scenarios like e-health, smart transportation, smart cities, assisted living, etc.

As we are currently living in the era of big data age, we have to seriously think about our data privacy and security when it comes to collating and accessing data from different interconnected IoT environments. In recent years, we have been encountering privacy violations and security breaches in the social IoT platforms [53,54]. We have to pay attention to data breach cases and privacy threats before we extensively use IoT applications and enjoy the benefits of the modern inventions. We have to prepare ourselves according to manage such privacy cases and increase privacy-awareness for our connected world.

Recently, the adoption of cloud and IoT in the healthcare field has been significantly improved health services and contributed to its innovation. Over the last few years, fog computing has emerged as the next level of computing that uses a considerable amount of storage and manage data at the edge of the network, including one or more near-edge devices [22,23]. As such, it is possible to localized computing infrastructures in which applications and data can be distributed in a more efficient and logical way between the data storage (e.g., cloud servers) and the users.

Recent effort has been put into combining IoT and cloud environments [1] in order to access data from the IoT sensors and relevant smart spaces. In the future, further investigation is required to build essential CAAC solutions by reasoning IoT-based contexts from different sensors. Such IoT contexts can be used for further decision-making process, like as follows.

- Deducing the daily living activities of elderly people.
- Health data access from IoTs and treatment progress monitoring.

### 6.2. An Emerging CAAC Mechanism

We present a new generation of fog-based CAAC mechanism for cloud and IoT-based data resources. We briefly describe its basic building blocks in this section. Our proposed CAAC mechanism addresses the above-mentioned security and privacy issues to some extent.

**Figure 4:**
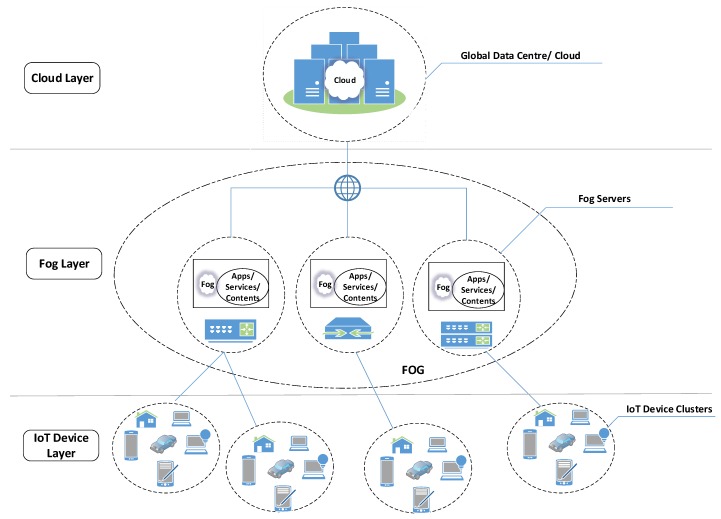
A new generation of the fog-based CAAC mechanism.

Figure 4 illustrates the proposed Fog-Based CAAC (FB-CAAC) framework, which has three layers: cloud, fog and IoT device layers.

#### 6.2.1. IoT Device Layer

The outermost layer of the IoT-Fog-Cloud architecture is IoT device layer which consists of a collection of IoT devices. The internet of things is a network of many devices, objects or physical things such as sensors, mobile phones, laptops, actuators, RFID tags and so on. Any object with embedded micro-controller and communication capabilities have the potential to be grouped into logical or geographical clusters. In a few years from now, dozens of billions of “things” will be connected [1]. As the number of connected devices is increasing, the rate to process and transmit the data also increases. IoT lacks computation and storage infrastructure and hence is usually integrated with the cloud to bridge this gap. IoT devices are highly heterogeneous at computing capacity, data communication protocol and mobility, which makes IoT device management and data communication challenging. Hence, to fulfil this requirement, two computing paradigms such as cloud and fog can be utilized together.

#### 6.2.2. Fog Layer

Fog computational nodes are known for providing localization and thus enabling low latency and context awareness, however global centralization is provided by the cloud. The fog nodes will be registered under the cloud’s authority at the closer to the end-users and will be responsible to provide services to the end-users (i.e., IoT devices).

The fog nodes/servers will act as ubiquitous tools and perform the integration of data that are coming from multiple, distributed cloud sources. A fog node can reduce the amount of data to be sent to the cloud and carries out the possible amount of data storage, controlling and computing at or near the end-user rather in remote cloud data centres. These fog nodes can fill the gap between IoT devices and cloud by enabling continuous service and also overcoming the latency and processing overheads by moving the execution of application logic from the cloud levels to intermediary levels at the edge of the networks.

#### 6.2.3. Cloud Layer

The infrastructure discussed in this paper consists of three layers composing of fog and cloud to support IoT applications as shown in Figure 4. While IoT devices are at the edge of the network, fog devices are distributed from IoT devices’ access point which leaves the cloud further away from the IoT devices. The IoT sensors or devices require requests from cloud services that again need to traverse through the fog and back to access the cloud’s resources.

Because of the special infrastructural requirements such as storage, power, space, qualified workforce and associated management costs, cloud data centres are usually deployed in a limited number of locations. The cloud providers will be responsible for registering end-users and fog servers.

IoT devices which are treated as end-users, cloud data centres can be at different distributed places. As IoT devices are scattered from cloud centres which can result in several challenges like high response time, heavy load on cloud and lack of global mobility. Moreover, existing cloud-based security services focus on redirecting web traffic to cloud for threat detection, and redirecting access control requests to the cloud for authorization [127]. This sounds impractical due to the growing number of devices and every device will need to connect to the cloud and update its security credentials every time there is a change.

Many devices operating in unprotected environments can be used to send false information [128]. Attackers can also damage the physical equipment, managing the messages to and from the system appearing to be normal. The solution to this is to move security measures to fog and let fog take decision on who can access resources in the cloud.

#### 6.2.4. Cloud-Fog Interplay

The cloud-based architecture will be extended by introducing an intermediate layer called “cloud-fog interplay” between IoT devices and cloud server. The aim of this is to provide higher response time, lower latency, higher security and smooth service delivery.

The emerging fog paradigm is expected to bring cloud-like services closer to the end-users’ proximity. This upcoming cloud-fog interplay will grant service providers with more degrees of freedom in terms of implementation and management resulting either in improved quality of service (QoS) or with the quality of experience (QoE) [129].

Applications dealing with big data and analytics requires both fog localization and cloud globalization. Smart grid [130] can be considered as a good example to illustrate cloud-fog interplay because of its different data hierarchies.

#### 6.2.5. An Access Request Using FB-CAAC in the IoT Scenario

This section presents a discussion about how to implement the proposed FB-CAAC framework when there is a data access request from an IoT device.

Let us consider an access request when an IoT device requests for any data resources. The fog nodes will process the request or forward it to the cloud or to other fog in the same domain. The cloud node can then process the request and send the response back to the IoT devices. The aim is to minimize service delays and to provide maximum security to IoT devices using a fog layer.

In the smart grid, data generated by grid devices and sensors which might require real-time processing can be fed to fog collectors at the edge of the network. This fog collectors can then process the data, filter the data if needed and send the rest of the data to higher tiers for visualization and reporting. These interactions can range anywhere from seconds to minutes to days, which makes it necessary for fog nodes to support some types of storage.

#### 6.2.6. Discussion and General Requirements

From our analysis of the context-sensitive access control literature [23], based on the identified challenges and open research issues, and focusing the high-level requirements for our proposed FB-CAAC mechanism, there is still a gap relating to the data access from distributed cloud environments utilizing existing access control solutions and consequently providing integrated results (e.g., an integrated data view from multiple data centres) to the end-users. Such a gap raises several general requirements (see the requirements from ‘R1’ to ‘R5’ in Table 6).

## 7. Conclusions

Over the last few decades, the continuous development of Internet-based technologies has produced an overwhelming flow and storage of data in the cloud. Due to the latency and processing overheads involved in managing and accessing such big data from distributed cloud data centres, currently, the organizations have been seeking appropriate access control solutions. On the one hand, they want to make the best use of such data, while on the other hand, they want to meet the privacy and security requirements of the different stakeholders. The result of this is the appearance of a clear gap between the existing context-aware access control (CAAC) mechanisms and the capacity of such traditional CAAC solutions to manage and control information and data resources in the cloud.

In this paper, we have reviewed various CAAC approaches and frameworks that have been applied to support access control to data and information resources at different granularity levels based on the dynamically changing contextual conditions. The contributions of this survey are as follows. We have made available a consolidated view of the wide range of access control-specific contextual conditions and provided an extensive study of the existing body-of-the-knowledge on CAAC mechanisms. In order to realize a new generation of CAAC model for cloud and fog networks, we have identified existing research issues and challenges based on some real-world application scenarios and case studies. Finally, we have proposed a new generation of Fog-Based CAAC (FB-CAAC) framework for accessing data from distributed cloud data centres along with IoT devices and fog computational nodes. In particular, the main contributions of this paper are listed as follows.

- Different taxonomies of contextual conditions and authorization models.
- An empirical analysis of the existing access control mechanisms.
- Opportunities, challenges and new directions of future research for cloud and fog networks.
- A future trend of emerging fog-based context-aware access control model.

This sweeping view on the analysis of the existing CAAC approaches and frameworks along with our new generation of FB-CAAC proposal that will help the engineers and scientists to investigate the new possibilities for further research directions, which are not still been well aligned with current state-of-the-art access control research.

## Figures and Tables

**Table 1:** The scope and contributions of the study.

Scope	Description
S1	We first cover the background of the traditional access control and context-aware access control literature.
S2	We then present different taxonomies of contextual conditions and authorization models according to the access control-specific contextual entities.
S3	We cover the existing context-sensitive access control approaches, including the Role-Based Access Control (RBAC) mechanisms and Context-Aware Access Control (CAAC) approaches for IoT sensor networks, privacy-preserving techniques and applications for distributed cloud databases and the policy-aware deployment and management of cloud applications.
S4	We divide the context-aware access control literature into main two categories: the access control mechanisms for centralized networks and the access control mechanisms for decentralized cloud and fog networks.
S5	We provide a comparative analysis of the existing context-aware access control mechanisms. We highlight the limitations and shortcomings of these mechanisms that motivate us to develop a new CAAC framework for cloud and fog networks.
S6	We discuss the directions of future research along with practical case studies, including access management against identify thefts, safeguarding health data against data breaches, protecting banking customers against data breaches, and security and privacy of the internet of things. We also include the research challenges and opportunities in these directions.
S7	In addition, we propose a new generation of fog-based CAAC model for today’s cloud and fog networks, including a layer-based framework.
S8	From our analysis of the state-of-the-art access control literature and open research issues, finally we identify the general requirements of an emerging fog-based CAAC mechanism.

**Table 2:** Dynamic conditions and contextual entities.

Research	Context Definition	Entity
Dey et al. [60]	The context information can be seen as any information that can be used to characterize the situation of an entity (an entity is a person, a place or an object).	Person, Place and Object
Kayes et al. [62]	The context information can be seen as any information that can be used to characterize the state of the relevant access control-specific entities and the state of the relevant relationships between different entities (an access control-specific entity is a user, a resource or an environment).	User, Resource and Environment

**Table 3:** Definition of context information.

Research	Context Definition
Dey et al. [60]	General Context Definition in Pervasive Domain: Focusing the pervasive computing domain, the general context information can be categorized into three types: person, place and object-specific.
Kayes et al. [62]	General Context Definition in CAAC Domain: Focusing the access control domain, the context information can be categorized into three types: user, resource and environment-specific. Based on the access control literature, the context information also can be categorized into two types: basic context and derived context.
Kayes et al. [19]	Basic Context Definition in CAAC Domain: The basic context can be captured or sensed directly from the raw contextual facts, such as the location context can be captured from the raw location coordinates.
Kayes et al. [6]	Derived Context Definition in CAAC domain: The derived context can be inferred from the basic context information, such as derived or inferred contexts can be relationship-based, situational and fuzzy context.
Kayes et al. [20]	Relationship Context Definition in CAAC domain: The relationship context can be categorized as social or interpersonal relationship and location-specific or co-located relationship. The interpersonal relationship context can be inferred from the users’ profile context and the colocated relationship context can be derived from the users’ location context.
Kayes et al. [10]	Situational Context Definition in CAAC domain: A situational context is defined as the states of the access control-specific entities and the states of the relationships between such entities at a particular time that are relevant to a certain goal or purpose of a resource access request. The situation value can be obtained based on the access request (i.e., from the sensed contexts, and/or inferred contexts).
Kayes et al. [63]	Fuzzy Context Definition in CAAC domain: The fuzzy context information cannot be obtained directly from the raw contextual facts, which are the crisp sets, where the value can be ranged either 0 or 1. Such information can be obtained based on the degree of membership function, where the value can be ranged from 0 to 1, or based on another type of measure like low, medium or high. A patient’s health status is “70% critical with a critically level of 0.7 or high”, which is a fuzzy context.

**Table 4:** The access control research and contribution areas in the centralized networks.

Research	Contribution Areas
[15,16,17,67,68,69]	The RBAC Approaches with Spatial and Temporal Contexts
[6,19,25,62,71,72,73]	The RBAC Approaches with User, Resource, and Environment-Centric Contexts
[20,74,75]	The RBAC Approaches with Relationship Contexts
[8,10,21,64,76,77,78,79,80,81]	The RBAC Approaches with Situational Contexts
[7,63,82,83,84,85,86]	The RBAC Approaches with Fuzzy Contexts

**Table 5:** The access control research and contribution areas in the decentralized networks.

Research	Contribution Areas
[22,23,91,95,96,97,98]	The CAAC Approaches for Accessing Data from Edge, IoT and Cloud Networks
[99,100,101,102,103,104,105,106,107,108,109]	The Privacy-Preserving Protocols and Mechanisms for Distributed Cloud Databases
[110,111,112,113,114,115]	The Privacy-Preserving Mechanisms for Cloud Service Providers
[116,117,118,119,121,122,123]	The Policy-Aware Deployment and Management of Cloud Applications

**Table 6:** The general requirements of fog-based CAAC mechanism.

Requirement	Description
R1	How to capture and derive the relevant contextual conditions from the IoT, fog and cloud environments? Thus, there is a need for a generic context model to capture and represent relevant contextual conditions using information provided through IoT devices and the associated fog and cloud environments.
R2	How to effectively specify the context-aware access control policies to manage and control data from distributed cloud sources by means of reducing computational overheads? Towards this goal, we can model a single set of access control policies instead of multiple sets of policies for different data sources.
R3	In order to reduce the overheads, how to build a global data model to map the identical attributes (e.g., the contextual conditions) from the relevant data sources and apply the same set of policy in the intermediary fog layer for accessing data from multiple sources?
R4	Focusing on the privacy requirements of the multiple stakeholders, how the end-users can prevent unauthorized entities and can ensure the privileges to access only certain information except sensitive and personally identifiable information?
R5	In order to limit the permissions to data from multiple cloud centres and achieve trust among all peers (e.g., users and other stakeholders), how to build an appropriate data sharing mechanism for all the entities involved, like IoT devices, fog servers and cloud data centres.
